# Development of RT h-CLAT, a Rapid Assessment Method for Skin Sensitizers Using THP-1 Cells as a Biosensor

**DOI:** 10.3390/bios14120632

**Published:** 2024-12-20

**Authors:** Hiroki Koyama, Ayami Maeda, Peiqi Zhai, Keiichiro Koiwai, Kouichi Kurose

**Affiliations:** 1 Department of Food Science and Technology, Tokyo University of Marine Science and Technology, Tokyo 108-8477, Japan; hkoyam1@kaiyodai.ac.jp (H.K.); amssmkj.12popcorn@gmail.com (A.M.); saihaiki123@gmail.com (P.Z.); 2 Department of Marine Biosciences, Tokyo University of Marine Science and Technology, Tokyo 108-8477, Japan; koiwai@kaiyodai.ac.jp

**Keywords:** *HMOX1*, *JUN*, RNA-Seq analysis, alternative methods, in vitro skin sensitization test, biomarker

## Abstract

In recent years, in vitro skin sensitization assays have been recommended as animal-free alternatives for the safety assessment of cosmetics and topical drugs, and these methods have been adopted in OECD test guidelines. However, existing assays remain complex and costly. To address this, we recently developed a more efficient, cost-effective, and accurate method for evaluating skin sensitizers by using immune cell-derived THP-1 cells as a biosensor, coupled with an RT-PCR-based assay. In this study, we further refined this method to enable even faster assessment of skin sensitization. By performing comprehensive RNA sequencing (RNA-Seq) analysis, we examined gene expression profiles induced by sensitizers in THP-1 cells to identify potential sensitization markers, ultimately selecting the optimal markers and conditions for evaluation. Our findings indicate that after exposing a test chemical to THP-1 cells for 5 h, measuring the expression levels of the *JUN* and *HMOX1* genes via real-time PCR allows for a reliable assessment of sensitization. A test compound is defined as a sensitizer if either gene shows a more than two-fold increase in its expression compared to the control. Applying this improved method, designated as RT h-CLAT, we evaluated the sensitization potential of 43 chemicals. The results demonstrated higher accuracy compared to the human cell line activation test (h-CLAT) listed in the OECD guidelines, while also reducing the required assessment time from two days to one.

## 1. Introduction

To ensure our safety, it is essential to evaluate the skin sensitization potential of chemical components in cosmetics and topical medications, as these chemicals can cause allergic contact dermatitis through repeated localized exposure [1]. Historically, experimental animals were used to assess the sensitization of these chemicals, leading to the establishment of assessment guidelines [2,3,4]. However, beginning with EU regulations [5] and advancing globally, there has been growing advocacy for the development and sale of cosmetics free from animal testing. Today, many countries encourage, and in some cases enforce, bans on animal testing for cosmetics [6]. Consequently, alternative methods for sensitization evaluation not involving experimental animals, such as in vitro skin sensitization assays [7,8], have progressed. To develop these alternatives, it is crucial to understand the in vivo reactions that lead to dermatitis. The Organisation for Economic Co-operation and Development (OECD) has outlined four sequential key events involved in skin sensitization [9].

In the first key event (key event 1), skin sensitization begins when electrophilic substances form covalent bonds with nucleophilic sites in skin proteins [10]. During key event 2, in keratinocytes, the chemical hapten–protein complex triggers an inflammatory response, involving changes in gene expression associated with specific cellular signaling pathways, such as the antioxidant/electrophile response element-dependent pathway [11,12,13,14]. In key event 3, dendritic cell activation occurs, accompanied by increases in chemokines and cytokines known as co-stimulatory and intercellular adhesion molecules [15,16,17,18]. Finally, in key event 4, there is activation and proliferation of T cells [9].

One established method for evaluating skin sensitization in vivo is the mouse local lymph node assay (LLNA), which uses T-cell activation as an indicator [19,20]. Recognized as a reliable test for skin sensitization, this method has been incorporated into the OECD skin sensitization test guidelines [2,3]. However, due to the limitations associated with animal testing, several alternative non-animal test methods have since been developed. One such alternative, h-CLAT, is an in vitro sensitization assay that utilizes the human monocytic leukemia cell line THP-1, which exhibits similar reactivity to human dendritic cells in response to chemical substances [21,22,23,24]. h-CLAT, corresponding to key event 3 (dendritic cell activation), has been adopted by the OECD as an in vitro alternative assay.

In h-CLAT, sensitization potential is evaluated by quantifying changes in the surface expression of CD86 and CD54 on THP-1 cells exposed to test chemicals, using flow cytometry, and demonstrates reliability nearly equivalent to the mouse LLNA [7]. However, flow cytometers are costly and require complex handling. To overcome these limitations, we developed a novel testing method for sensitizers that utilizes real-time RT-PCR (henceforth referred to as modified h-CLAT) as an alternative to flow cytometry [25]. During the development of this method, we performed a comprehensive analysis of genes specifically induced in THP-1 cells 24 h after exposure to sensitizing substances. From this analysis, we identified *TREM1* and *TNFRSF12A* as sensitization marker genes. Ultimately, by measuring the expression levels of *TREM1* and *TNFRSF12A* in THP-1 cells 24 h after exposure to test chemicals using real-time PCR, and applying sensitization criteria, we successfully evaluated the sensitizing and non-sensitizing properties of all 13 tested chemicals accurately. Furthermore, this real-time PCR-based approach is not only accurate but also comparatively simpler and more cost-effective than h-CLAT.

In this study, we explored whether the exposure time of chemicals on THP-1 cells could be further reduced from 24 h to 5 h. Additionally, we identified optimal marker genes for our sensitization assessment method using RNA sequencing (RNA-Seq) analysis. Our novel method, named “RT h-CLAT”, overcomes the disadvantages of conventional methods, which are costly and time-consuming to operate. Moreover, the simplicity of operation is noteworthy. Our findings suggest that this approach could enable quicker assessment results and improve the efficiency of skin sensitization testing.

## 2. Materials and Methods

### 2.1. Materials

The sensitizing and non-sensitizing chemicals used in this study are listed in Tables S1–S3, along with their abbreviations, Chemical Abstracts Service (CAS) numbers, solvents, a concentration of 75% cell viability (CV75), skin sensitization potency categories in murine LLNA, judgements in h-CLAT, and manufacturer. The CV75 is the value calculated in the modified h-CLAT with 24 h chemical exposure [25], which is denoted as “24h CV75”. Almost all chemicals have been evaluated in the murine LLNA and h-CLAT cell lines which are in vivo and in vitro skin sensitization tests adopted as OECD guidelines, respectively [7,19,26,27]. Dimethyl sulfoxide (DMSO, Sigma-Aldrich Inc., St. Louis, MO, USA) and cell culture medium were used as solvents.

### 2.2. Cell Culture

THP-1, a human acute monocytic leukemia cell line, was purchased from the American Type Culture Collection (ATCC, Manassas, VA, USA). Cell culture was performed in accordance with the standard procedure described by the OECD Test Guideline 442E [7]. THP-1 cells were cultured in RPMI-1640 (Sigma-Aldrich Inc., St. Louis, MO, USA) supplemented with 10% heat-inactivated fetal bovine serum (Biosera, Cholet, France), penicillin-streptomycin-L-glutamine solution (100 unit/mL penicillin, 100 μg/mL streptomycin, 2 mM L-glutamine; FUJIFILM Wako Pure Chemical Co., Osaka, Japan), and 0.05 mM 2-mercaptoethanol (Sigma-Aldrich Inc. St. Louis, MO, USA) at 37 °C in a humidified atmosphere of 5% CO_2_ and 95% air. The expanded cells were frozen in aliquots. Each aliquot was thawed and used after 2 weeks for up to 2 months. The cells were routinely passaged every 48–72 h at a density of 0.2–0.3 × 10^6^ cells/mL.

### 2.3. Chemical Treatment of THP-1 Cells for RNA-Seq Analysis

We used nine reference chemicals, 2,4-dinitrochlorobenzene, 1,4-phenylendiamine, nickel sulfate, 2-mercaptobenzothiazole, R(+)-limonene, imidazolidinyl urea, isopropanol, glycerol and 4-aminobenzoic acid, for chemical treatment (Table S1). These chemicals are 9 of the 10 recommended substances for demonstrating technical proficiency with the h-CLAT assay listed in the OECD Test Guideline 442E [7]. The remaining compound, lactic acid (a non-sensitizer), was excluded due to technical challenges. Specifically, treatment with lactic acid consistently resulted in poor RNA yield and quality, likely due to unknown effects on cellular processes or culture conditions. This limitation made it unsuitable for RNA-Seq analysis in this study. These chemicals were dissolved in DMSO or culture medium. After adjusting the concentrations of the chemicals to 24 h CV75, water-soluble and fat-soluble chemicals were diluted 50-fold and 250-fold, respectively, using culture medium. These dilutions were mixed with equal volumes of culture medium containing THP-1 cells and incubated for 5 h in a 24-well flat-bottom plate (1.0 × 10^6^ cells/mL/well, *n* = 3/dose) at 37 °C in a humidified atmosphere of 5% CO_2_ and 95% air. The same method was used to culture THP-1 cells in the medium with only chemical-free solvents added as a control. In this case, the final concentration of DMSO was set to be 0.2%.

### 2.4. RNA-Seq Analysis

After 5 h of the chemical exposure, THP-1 cells were collected by centrifugation at 130× *g* for 4 min. Total RNAs were extracted and purified from collected THP-1 cells using NucleoSpin RNA (Takara, Otsu, Japan), according to the manufacturer’s instructions. The purified total RNAs were quantified by Quantus Fluorometer (Promega, Madison, WI, USA) and QuantiFluor RNA System (Promega, Madison, WI, USA). Pair-end cDNA libraries were constructed from abovementioned total RNAs using NEBNext UltraTMII Directional RNA Library Prep Kit (New England Biolabs, Ipswich, MA, USA) and sequenced using NovaSeq 6000 (Illumina, San Diego, CA, USA) by Rhelixa (Tokyo, Japan).

The obtained data were mapped onto the Genome Reference Consortium Human Build 38 ver. 21 from GENCODE, which was used as the reference sequence, and transcripts per million (TPM) value was determined for each gene. Furthermore, to examine the effects of the administration of sensitizers, DEGSeq2 was used to find genes showing differences in the expression levels between the sensitizer group and the non-sensitizer group, between the sensitizer group and the control group, or between the non-sensitizer group and the control group. After omitting genes with fewer than 10 reads in all 11 test segments, including the control, log2FoldChange and adjusted *p*-value were obtained using DEGSeq2. The genes with |log2FoldChange| > 1 and adjusted *p*-value < 0.05 were selected as those whose expression levels were altered by the exposure of sensitizing substances.

### 2.5. Real-Time PCR Assay

Real-time PCR was performed in triplicate for the 10 genes (*HMOX1*, *JUN*, *PPP1R15A*, *ULBP2*, *SAT1*, *EGR1*, *GADD45B*, *PMAIP1*, *DDIT3* and *BTG2*) whose expression levels were up-regulated and the 2 genes (*ICMT* and *CCR2*) whose expression levels were down-regulated by exposure to sensitizers according to RNA-Seq analysis. Total RNAs were isolated from chemical-treated THP-1 cells using ISOGEN II reagent (Nippon Gene, Tokyo, Japan), according to the manufacturer’s instructions. cDNA was prepared from 250 ng of the total RNA using ReverTra Ace qPCR RT Master Mix with gDNA Remover (Toyobo, Osaka, Japan), according to the manufacturer’s instructions. Singleplex real-time quantitative PCR was performed on the PikoReal 96 Real-Time PCR system (Thermo Fisher Scientific, Waltham, MA, USA) in an 8.0 μL reaction mixture that contained 0.8 μL cDNA, 0.16 U uracil-DNA glycosylase (UNG), 0.4 pmol gene-specific primers, and 4 μL of THUNDERBIRD Next SYBR qPCR Mix (Toyobo, Osaka, Japan). The PCR conditions consisted of 25 °C for 10 min for UNG reaction and initial denaturation at 95 °C for 30 s followed by 40 cycles at 95 °C for 5 s and 60 °C for 30 s. At the end of amplification, melting curve analysis was performed from 65 °C to 95 °C to verify the specificity of the amplicons. After PCR, the quantification cycle (Cq) values were calculated by the PikoReal Software version 2.2 (Thermo Fisher Scientific, Waltham, MA, USA). The nucleotide sequences of the primers used in this qPCR are shown in Table 1. All primers were designed using Primer-BLAST (NCBI) based on the sequences in the NCBI genome database. The Cq values were normalized to the human glyceraldehyde-3-phosphate dehydrogenase (*GAPDH*) gene. The changes in the gene expression levels with the ratio of exposed samples to the solvent control samples were calculated by the comparative Cq method as fold changes [28,29].

### 2.6. Selection of the Candidate Marker Genes

Five genes (*HMOX1*, *JUN*, *PPP1R15A*, *PMAIP* and *BTG2*) that showed the similar changes in their expression levels by both RNA-Seq analysis and real-time PCR were selected as candidate marker genes. To examine whether exposure of 9 newly prepared chemicals (Table S2) to THP-1 cells for 5 h altered the expression levels of the abovementioned five genes, real-time PCR was performed. Furthermore, the expression levels of *HMOX1* and *JUN* genes were examined when THP-1 cells were treated with other 28 chemicals (Table S3) for 5 h. The same methods as in the above sections were used for chemical treatment of THP-1 cells and real-time PCR.

## 3. Results

### 3.1. Gene Expression Analysis

**Figure 1:**
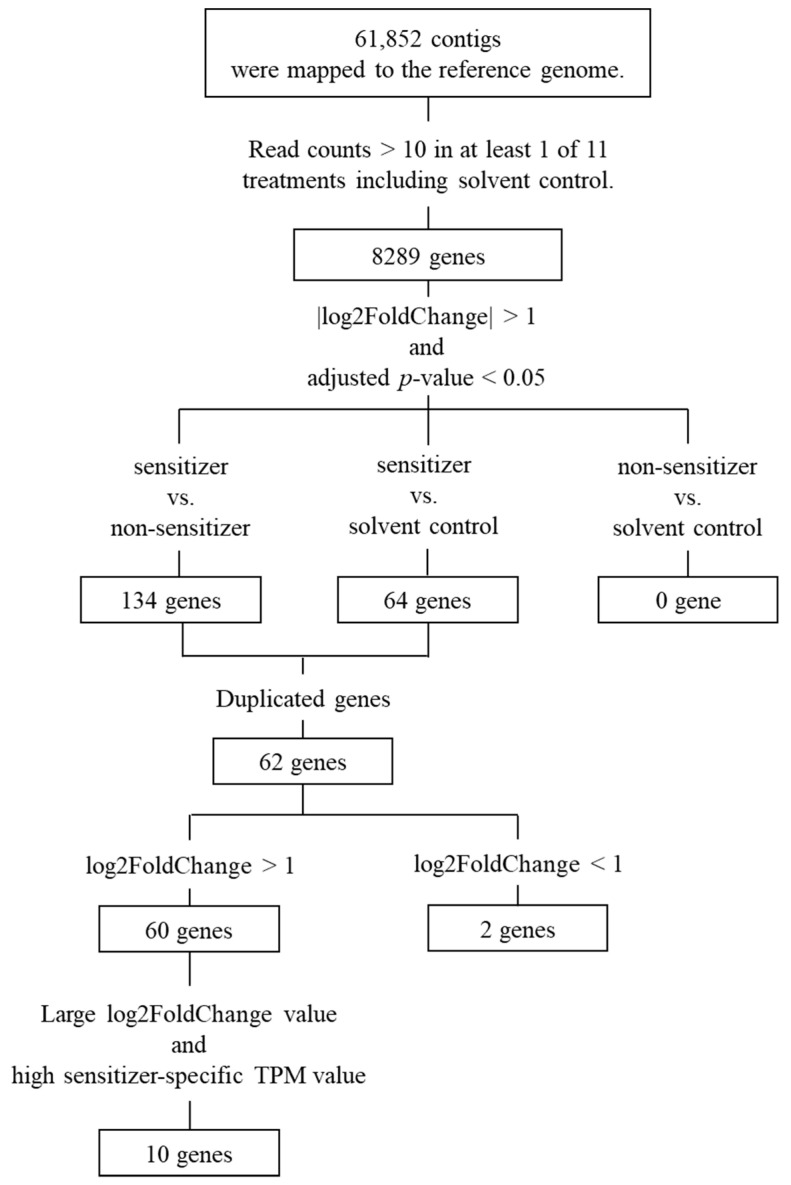
The overall process of selecting candidate marker genes for sensitization assessment using THP-1 cells. After selecting genes whose expression levels were significantly increased or decreased compared to the control, 12 genes have remained as candidates. The expression levels of 10 genes were increased by the treatment of sensitizers, while those of 2 genes were decreased.

The genes expressed in THP-1 cells exposed to nine different reference chemicals, including six sensitizers and three non-sensitizers (Table S1) for 5 h were examined by RNA-Seq analysis. These 9 chemicals were selected from the 10 recommended substances for demonstrating technical proficiency with the h-CLAT assay in OECD Test Guideline 442E [7]. Lactic acid was excluded due to technical challenges in isolating high-quality RNA after treatment, as detailed in Section 2.3. The analysis revealed an average of approximately 37 million reads per one treatment in the total 11 treatments, including two types of controls with no additives and only DMSO (solvent), and, as shown in Figure 1, these reads were mapped to 61,852 regions of reference genome. Furthermore, 8289 genes had read counts of more than 10 in any of the assays.

**Figure 2:**
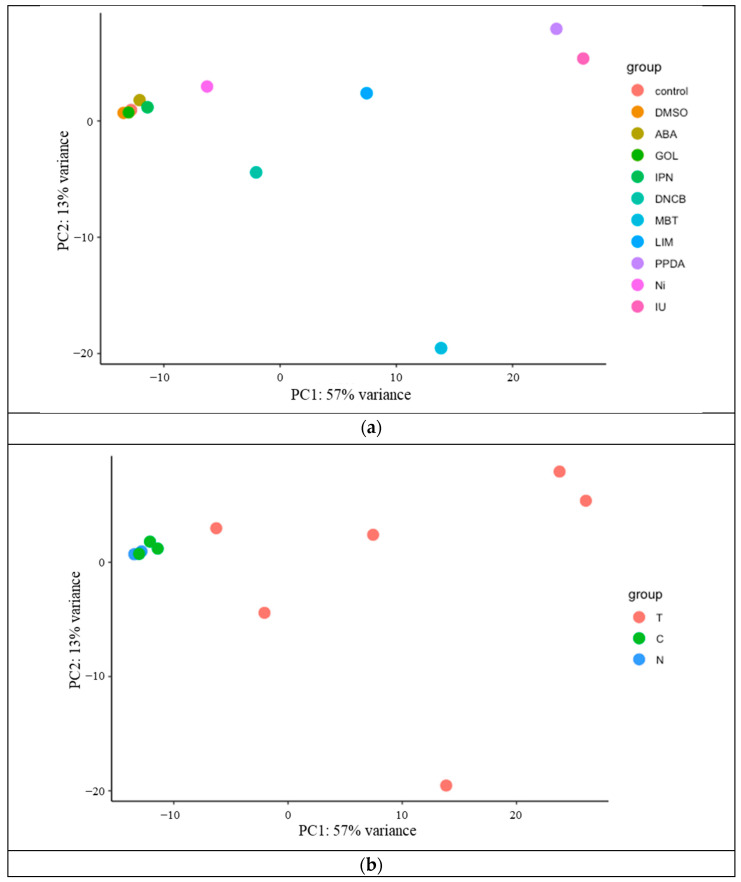
Principal component analysis of genes expressed in THP-1 cells exposed to sensitizers and non-sensitizers. PC1 and PC2 indicate the first and second principal component scores, respectively. The number shows the contributing ratio of each score. (**a**) Each circle shows the expression profile of genes in THP-1 cells exposed to different chemicals including the control. (**b**) Each circle shows the expression profile of genes in THP-1 cells exposed to sensitizers (T), non-sensitizers (C), and controls (N).

A principal component analysis was performed on these genes, and the results are shown in Figure 2a,b. Figure 2a shows that the gene expression patterns differed depending on the treated chemicals. In more detail, the groups treated with non-sensitizing chemicals and controls exhibit almost the same expression patterns (Figure 2b). In contrast, when treated with sensitizing chemicals, gene expression patterns differed depending on chemicals (Figure 2b).

There were 62 genes out of 8289 genes whose expression levels altered only with the treatment of sensitizing chemicals under parameters |log2FoldChange| > 1 and adjusted *p*-value < 0.05 (Tables S1 and S2). Sixty of these genes were up-regulated, and the remaining two were down-regulated. On the other hand, no differences were observed between the non-sensitizing chemical treated group and the control group under parameters |log2FoldChange| > 1 and adjusted *p*-value < 0.05.

Among the 60 genes whose expression levels were up-regulated by the sensitizing chemical treatment, 10 genes (*HMOX1*, *JUN*, *PPP1R15A*, *ULBP2*, *SAT1*, *PMAIP1*, *GADD45B*, *DDIT3*, *BTG2* and *EGR1*) were selected as those with large log2FoldChange values and high TPM values. Two genes (*ICMT* and *CCR2*) whose expression levels were down-regulated by sensitizing chemical treatment were also selected. The overall process for selecting these 12 genes is shown in Figure 1. Log2FoldChange values and the statistical data for the selected 12 genes are shown in Tables S4 and S5, and TPM values for the 13 genes, including the internal control *GAPDH*, are shown in Table S6.

### 3.2. Selection of the Candidate Marker Genes

The relative expression levels of 12 candidate marker genes (*HMOX1*, *JUN*, *PPP1R15A*, *ULBP2*, *SAT1*, *PMAIP1*, *GADD45B*, *DDIT3*, *BTG2*, *EGR1*, *ICMT* and *CCR2*) were examined by real-time PCR, using the *GAPDH* gene as an internal control. Table 2 presents the fold changes in the expression levels of the candidate marker genes by chemical exposure relative to those in the controls using only solvents. In addition, Table 2 includes the fold changes in the candidate marker genes based on TPM values obtained from RNA-Seq analysis. If there was a two-fold or greater differences between the gene expression level obtained from real-time PCR and that obtained from RNA-Seq analysis in two or more of the three assays, the results of real-time PCR and RNA-Seq analysis were judged to be inconsistent. Applying this criterion, five (*HMOX1*, *JUN*, *PPP1R15A*, *BTG2* and *PMAIP1*) of the 12 genes were consistent in the results between real-time PCR and RNA-Seq analysis (Table 2). Therefore, these five genes were selected as the new candidate marker genes to evaluate sensitization.

### 3.3. Evaluation of New Candidate Marker Genes

**Figure 3:**
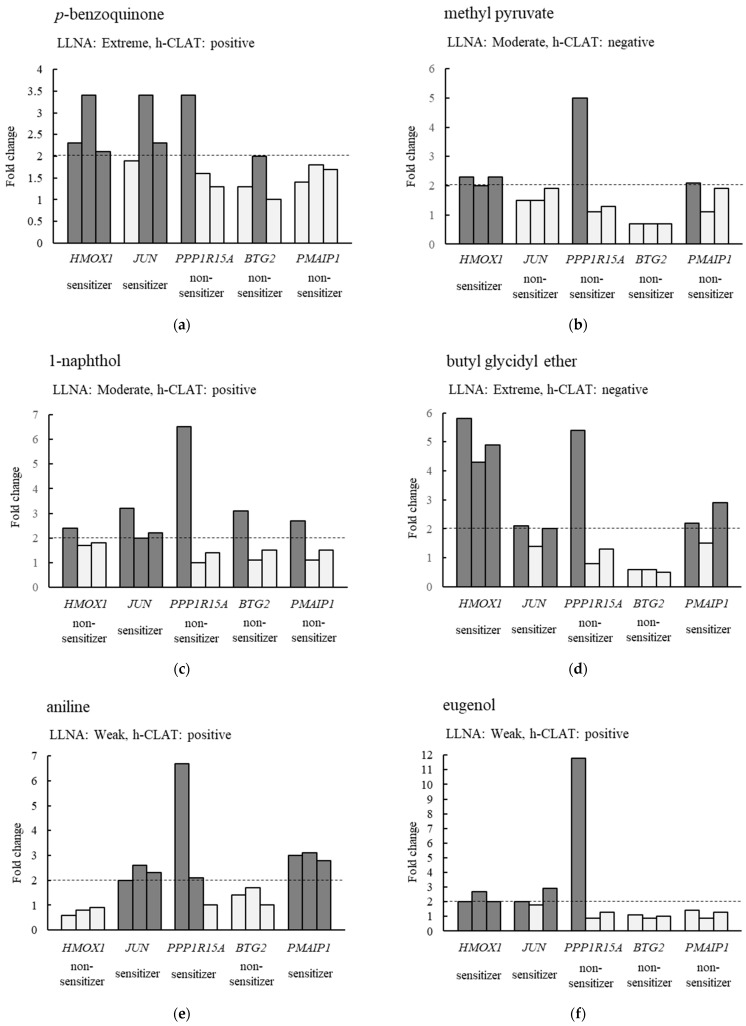

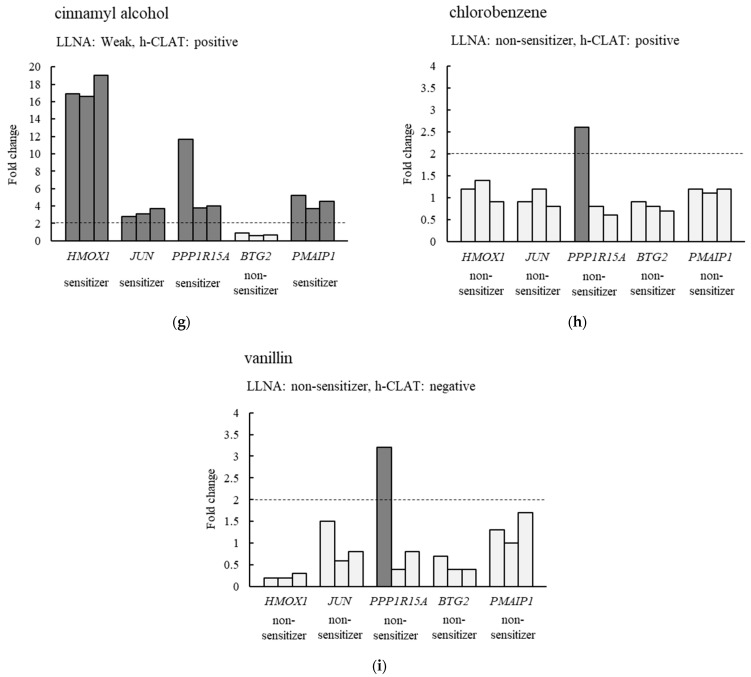
Changes in the expression levels of five candidate marker genes after chemical treatments to THP-1 cells. (**a**–**i**) The changes in the expression levels of *HMOX1*, *JUN*, *PPP1R15A*, *BTG2*, and *PMAIP1* genes after treatment of THP-1 cells with *p*-benzoquinone, methyl pyruvate, 1-naphthol, butyl glycidyl ether, aniline, eugenol, cinnamyl alcohol, chlorobenzene, and vanillin, respectively. The results of LLNA [3,19] and h-CLAT [7,26] for each chemical are also shown. Dark gray bars indicate more than 2-fold increases in the gene expression levels compared to the control. If the expression level of the gene was increased more than 2-fold in two or more of the three trials, the chemical used in the treatment was evaluated as a sensitizer.

The expression levels of five genes (*HMOX1*, *JUN*, *PPP1R15A*, *BTG2* and *PMAIP1*) were increased by treatment with six sensitizers (DNCB, PPDA, NiSO_4_, MBT, LIM and IU). Therefore, we investigated whether other sensitizers also caused changes in the expression levels of these five genes. Figure 3 and Table S7 show the fold changes in the expression levels of the above-mentioned five genes in THP-1 cells treated with the nine chemicals listed in Table S2. First, we examined whether the expression level of the focused gene increased more than 2-fold in response to the chemical treatment. If a 2-fold or more increase in the expression level was observed in two or more of the triplicate real-time PCR assays, it was determined that the treated chemical was a sensitizer. Applying this criterion to *HMOX1*, the results for seven out of nine chemicals were consistent with the murine LLNA Category (Figure 3 and Table S7). Considering the other genes, eight out of nine chemicals for *JUN*, four out of nine chemicals for *PPP1R15A*, two out of nine chemicals for *BTG2* and five out of nine chemicals for *PMAIP1* were consistent with the murine LLNA Category. No matter which gene was used as a marker, there were chemicals whose sensitization assessment was not consistent with the murine LLNA Category. However, if either one of *HMOX1* or *JUN* exceeded the criteria, the test chemicals were classified as a sensitizer, resulting in all assessments of the nine chemicals being consistent with the murine LLNA Category (Figure 3 and Table S7). In addition, when sensitization was evaluated for the 28 chemicals listed in Table S3, using both *HMOX1* and *JUN* as markers, the results were consistent with the LLNA Category for 23 chemicals (Table 3).

## 4. Discussion

h-CLAT is a good method for assessing chemical sensitization because it does not use animals and has a relatively high match rate of 85% with murine LLNA results [7]. In addition, h-CLAT has the advantage of lower costs and shorter testing periods compared to murine LLNA; however, it has the disadvantage of being complicated to operate. Thus, the modified h-CLAT was developed as a simpler method in our previous study [25]. This method focuses on the genes whose expression levels are increased in THP-1 cells exposed to sensitizers for 24 h and real-time PCR is used to evaluate sensitization. In the present study, we examined whether sensitization assessment is possible in the modified h-CLAT even when the chemical exposure time is reduced from 24 h to 5 h.

First, it is necessary to search for marker genes whose expression levels change in response to the exposure of sensitizers. Arkusz et al. identified genes whose expression levels altered in DC cells and applied them as marker genes to the assay using THP-1 cells, resulting in less accuracy in the evaluation of sensitizers [30]. Thus, we performed RNA-Seq analysis to search for genes whose expression levels are altered in THP-1 cells exposed to sensitizers. RNA-Seq analysis revealed that the expression levels of 62 genes changed in a sensitizer-specific manner regardless of the reduction in the exposure time of the chemicals to THP-1 cells to 5 h (Tables S8 and S9). This indicates that the changes in the gene expression levels occur specifically in the sensitizer-treated group even when the exposure time is as short as 5 h. Furthermore, 10 genes (*HMOX1*, *JUN*, *PPP1R15A*, *ULBP2*, *SAT1*, *EGR1*, *GADD45B*, *PMAIP1*, *DDIT3* and *BTG2*) with high expression levels and a large rate of increase in their expression levels and 2 genes (*ICMT* and *CCR2*) exhibiting a decrease in their expression levels were selected as candidate markers (Table S4). In particular, *HMOX1* and *JUN*, whose expression levels increased greatly (log2FoldChange > 5), were also up-regulated in a sensitizer-specific manner in the modified h-CLAT [25]. Moreover, it has also been reported that the expression level of *HMOX1* gene increased after the exposure of sensitizers to CD34-DC [31,32]. These results suggest that *HMOX1* is a potential candidate marker gene for sensitization assays.

Of the above 12 candidate marker genes, there was a consistency between the results of RNA-Seq analysis and those of real-time PCR with respect to the changes in the expression levels of 5 genes (*HMOX1*, *JUN*, *PPP1R15A*, *BTG2* and *PMAIP1*). Although the expression levels of these five genes were specifically increased for the sensitizers recommended by the OECD [7], further studies were needed to determine whether they can actually be used for sensitization assessment. Thus, the expression levels of five candidate marker genes were quantified using real-time PCR for THP-1 cells exposed to an additional nine chemicals. When a 1.5-fold or greater increase in the expression level was defined as the exposed chemical being sensitizing, only *JUN* was able to correctly evaluate all 18 chemicals. Therefore, *JUN* was considered to be an optimal marker gene for short-term chemical exposure. However, it has been suggested that more reliable results can be obtained by using multiple markers in evaluation assays where protein or gene expression levels are used as indicators such as h-CLAT and modified h-CLAT [25,33]. After searching for a marker gene that could be used in combination with *JUN*, *HMOX1* was determined to be appropriate. On the other hand, we have reported that *TREM1* and *TNFRSF12A* are the best marker genes for THP-1 cells exposed to chemicals for 24 h [25]. Our present study revealed that the marker genes differed depending on the exposure time of chemicals.

HMOX1 is a crucial enzyme involved in the degradation of heme into biliverdin, free iron, and carbon monoxide. It plays a significant role in cellular defense mechanisms against oxidative stress and inflammation [34]. The upregulation of *HMOX1* in response to oxidative stress is well-documented, and it is known to be involved in various signaling pathways, including those related to immune responses and inflammation [35]. In the context of skin sensitization, *HMOX1* has been shown to be upregulated in response to contact sensitizers in dendritic cells and the THP-1 cell line, suggesting its involvement in the cellular response to sensitizing agents [31]. Specifically, the Keap1/Nrf2 pathway, which regulates the expression of *HMOX1*, is activated by electrophilic molecules, including sensitizers, leading to increased expression of *HMOX1* [31]. This pathway’s activation indicates that *HMOX1* could serve as a biomarker for the detection of sensitization potential of chemicals. To date, several studies have reported the upregulation of *HMOX1* in THP-1 cells upon exposure to sensitizing agents. For instance, Ade et al. (2009) demonstrated that *HMOX1* expression is significantly increased in THP-1 cells treated with various contact sensitizers [31]. Additionally, Zhong et al. (2018) highlighted the role of HMOX1 in skin sensitization, proposing that its induction is a consistent marker for skin sensitizers [36].

JUN, a component of the AP-1 transcription factor, is involved in regulating gene expression in response to a variety of stimuli including stress, cytokines, and growth factors [37]. JUN plays a pivotal role in cellular processes such as proliferation, differentiation, and apoptosis. In terms of skin sensitization, JUN is implicated in the cellular response to sensitizers through its role in the regulation of inflammatory and immune responses. The induction of JUN can lead to the expression of various cytokines and chemokines that are crucial for the development of allergic contact dermatitis. Although the specific pathways through which JUN contributes to skin sensitization are not fully elucidated, its involvement in the broader context of immune and inflammatory responses supports its relevance as a marker [38]. The selection of *HMOX1* and *JUN* as markers for our rapid assessment method is based on their significant roles in the cellular response to sensitizers and their consistent upregulation in THP-1 cells upon exposure to these agents. While the exact mechanisms by which these genes contribute to skin sensitization are not entirely clear, the experimental data strongly support their use in our method. Further research is needed to fully understand the pathways and mechanisms involved, but the current evidence underscores their potential as reliable markers for skin sensitization.

In our present study, we decided to evaluate the exposed chemicals as sensitizing if the expression levels of either *JUN* or *HMOX1* increased more than 2-fold. To confirm how effective the above criteria are, we performed sensitization assays on a further number of additional chemicals. As a result, 39 of the 43 chemicals used in this study were evaluated correctly (Table 3). Furthermore, the h-CLAT was able to correctly evaluate 36 of the 43 chemicals (Table 3), indicating that the method used in this study named RT h-CLAT was more accurate. RT h-CLAT, like the conventional h-CLAT method, is a binary assessment method that determines whether a chemical is sensitizing or non-sensitizing. It cannot evaluate the degree of sensitization, such as categorizing chemicals as moderate, strong, or extreme sensitizers, as is possible with the LLNA. A sensitization assay of sodium sulfite, which has been reported to induce food allergy [39], also showed a more than 3-fold increase in the expression level of the *JUN* gene (Table 3). This suggests that RT h-CLAT can be used to evaluate the sensitization not only to chemicals that induce skin sensitivity but also to those that induce food allergy.

It is important to note that, like other in vitro skin sensitization tests, RT h-CLAT cannot perfectly replicate in vivo results. The h-CLAT, for instance, shows an 85% concordance rate with murine LLNA results [25]. In the present study, while RT h-CLAT correctly evaluated 39 of the 43 chemicals tested, one notable discrepancy involved potassium dichromate, which was categorized as an “extreme” sensitizer by LLNA but under-predicted as a non-sensitizer by RT h-CLAT (Table 3). Conversely, RT h-CLAT showed distinct advantages in other cases. For example, benzoyl peroxide, classified as an “extreme” sensitizer by LLNA but predicted as negative by h-CLAT, was correctly identified as a sensitizer by RT h-CLAT (Table 3). This suggests that RT h-CLAT offers advantages in some cases, highlighting its potential utility. Moreover, the OECD guidelines for skin sensitization recommend considering test methods that reflect at least two of the first three key events in the Adverse Outcome Pathway for sensitizers, as consistency across multiple results enhances reliability [40]. Based on this, combining RT h-CLAT with other animal-free assays [8,41] could potentially enable more robust and reliable evaluations.

The RT h-CLAT method described in this study possesses several advantages over the conventional h-CLAT method currently validated under OECD Test Guideline 442E [7]. These include faster assessment times, simpler operation, and the feasibility of using standard laboratory equipment. Despite the challenges associated with regulatory validation and adoption, we believe that the scientific merit and practical benefits of RT h-CLAT justify efforts toward its establishment as a validated test guideline in the future.

## 5. Conclusions

We have developed a method named RT h-CLAT to assess the sensitization potential of chemicals in a shorter time by using THP-1 cells as a biosensor. RT h-CLAT involves the exposure of test chemicals to THP-1 cells for 5 h, followed by measuring the expression levels of *HMOX1* and *JUN* genes using real-time PCR. If the expression of either *JUN* or *HMOX1* increases at least two-fold, the test chemical is assessed as having sensitization potential. This approach is simpler, more cost-effective [25], more accurate, and more time-efficient compared to conventional methods such as h-CLAT, which require the use of a flow cytometer. Thus, our cell-based real-time PCR assay using THP-1 cells as a biosensor has the potential to become a major method for evaluating the skin sensitization potential of chemicals.

## Figures and Tables

**Table 1:** Gene name and primer sequences used in real-time PCR.

Gene Name	Gene Symbol	Sense Primer (5′-3′)	Antisense Primer (5′-3′)
*Heme oxygenase 1*	*HMOX1*	TGAACTCCCTGGAGATGACTC	AGCTCCTGCAACTCCTCAAA
*Jun proto-oncogene, AP-1 transcription factor subunit*	*JUN*	CAAGAACTCGGACCTCCTCA	CCGTTGCTGGACTGGATTAT
*Protein phosphatase 1 regulatory subunit 15A*	*PPP1R15A*	GAGGGCAGGGAAGTCAATTT	TCCTCCCCTGGGTTCTTATC
*BTG anti-proliferation factor 2*	*BTG2*	TGAGGTGTCCTACCGCATT	CACTTGGTTCTTGCAGGTGA
*DNA damage inducible transcript 3*	*DDIT3*	AGCAGAGGTCACAAGCACCT	CCTGGTTCTCCCTTGGTCTT
*Early growth response 1*	*EGR1*	CTTCGCTAACCCCTCTGTCT	TTGATGAGCTGGGACTGGTA
*Growth arrest and DNA damage inducible beta*	*GADD45B*	CAGAAGATGCAGACGGTGAC	AACTTGGCCGACTCGTACAC
*Phorbol-12-myristate-13-acetate-induced protein 1*	*PMAIP1*	CCGGCAGAAACTTCTGAATC	ACGTGCACCTCCTGAGAAAA
*Spermidine/spermine N1-acetyltransferase 1*	*SAT1*	GCAGCATGCACTTCTTGGTA	TCCAACCCTCTTCACTGGAC
*UL16 binding protein 2*	*ULBP2*	CCCCTGGGGAAGAAACTAAA	CTGAATGTCACGCAGTTGCT
*C-C motif chemokine receptor 2*	*CCR2*	ACCAGTCAACTGGACCAAGC	TGAACTTCTCCCCAACGAAG
*Isoprenylcysteine carboxyl methyltransferase*	*ICMT*	GTTTCGGCATCCTTCTTACG	CACTGTCAGGGCATAGCTGA
*Glyceraldehyde-3-phosphate dehydrogenase*	*GAPDH*	AGCCACATCGCTCAGACAC	GCCCAATACGACCAAATCC

**Table 2:** Changes in the expression levels of candidate marker genes by RNA-Seq analysis and real-time PCR.

**Chemical**	**Murine LLNA Category**	**h-CLAT Judgment**	***GAPDH***	***HMOX1***			***JUN***			***PPP1R15A***			***BTG2***			***DDIT3***			***EGR1***		
			**NGS**	**NGS**	**PCR**	**Judgment**	**NGS**	**PCR**	**Judgment**	**NGS**	**PCR**	**Judgment**	**NGS**	**PCR**	**Judgment**	**NGS**	**PCR**	**Judgment**	**NGS**	**PCR**	**Judgment**
2,4-Dinitrochlorobenzene	Extreme	p	1.2	5.2	3.2		7.7	9.3		9.3	8.0		3.3	30.7		1.6	1.4		5.4	8.3	
					3.2	Match		10.0	Match		21.1	Match		4.3	Match		0.7	Match		8.1	Match
					2.9			10.0			5.9			2.8			1.4			8.3	
1,4-Phenylendiamine	Strong	p	0.9	83.7	66.3		27.2	19.2		25.6	12.1		10.1	15.7		6.1	5.1		5.1	14.9	
					69.6	Match		22.8	Match		19.2	Match		16.7	Match		5.5	Match		16.4	Mismatch
					61.0			19.2			38.9			28.8			9.6			15.3	
Nickel sulfate	Moderate	p	1.1	3.4	2.3		4.5	2.8		3.1	2.2		4.6	3.9		2.4	1.5		1.1	1.3	
					2.3	Match		3.0	Match		2.0	Match		6.8	Match		2.2	Match		1.1	Match
					2.6			3.1			4.8			5.4			3.9			1.0	
2-Mercaptobenzothiazole	Moderate	p	1.1	63.7	39.7		25.1	27.6		12.3	10.1		3.8	9.3		27.2	33.4		3.2	3.9	
					37.3	Match		24.9	Match		27.3	Match		6.1	Match		11.6	Mismatch		3.6	Match
					22.5			25.4			21.7			5.2			63.1			3.4	
R(+)-Limonene	Weak	p	1.1	61.5	62.2		28.1	54.2		9.1	10.1		3.5	11.1		8.6	9.8		18.6	50.9	
					46.9	Match		54.9	Match		29.0	Match		4.6	Match		7.4	Match		36.5	Match
					54.6			63.6			17.8			6.1			18.8			32.9	
Imidazolidinyl urea	Weak	p	1.2	7.4	3.9		80.0	65.3		15.6	10.9		13.5	15.8		2.5	2.2		8.3	10.9	
					4.1	Match		46.8	Match		13.2	Match		18.6	Match		4.6	Match		8.3	Match
					4.2			51.9			17.9			24.1			3.3			8.6	
Isopropanol	non-sensitizer	n	1.0	1.3	1.6		1.4	0.8		1.2	1.1		1.4	1.6		1.7	2.3		0.6	0.8	
					1.5	Match		0.7	Match		0.9	Match		1.5	Match		3.3	Match		0.6	Match
					1.5			0.9			1.8			1.6			3.7			0.5	
Glycerol	non-sensitizer	n	1.0	1.5	1.4		1.2	0.8		1.0	1.0		0.9	1.4		1.1	1.1		1.1	1.6	
					1.6	Match		1.2	Match		1.0	Match		1.3	Match		1.2	Match		1.0	Match
					0.9			0.8			1.1			0.8			1.6			1.0	
4-Aminobenzoic acid	non-sensitizer	n	1.1	1.3	0.8		0.6	0.8		1.0	0.8		1.4	1.7		0.9	0.8		0.5	0.8	
					0.8	Match		0.6	Match		2.0	Match		1.1	Match		0.3	Match		0.5	Match
					0.9			0.8			0.9			4.5			1.2			0.6	
**Chemical**	**Murine LLNA Category**	**h-CLAT Judgment**	***GAPDH***	***GADD45B***			***PMAIP1***			***SAT1***			***ULBP2***			***CCR2***			***ICMT***		
			**NGS**	**NGS**	**PCR**	**Judgment**	**NGS**	**PCR**	**Judgment**	**NGS**	**PCR**	**Judgment**	**NGS**	**PCR**	**Judgment**	**NGS**	**PCR**	**Judgment**	**NGS**	**PCR**	**Judgment**
2,4-Dinitrochlorobenzene	Extreme	p	1.2	7.3	8.1		7.7	6.9		2.4	0.5		7.1	1.6		0.1	0.2		0.5	0.5	
					7.4	Match		6.5	Match		16.6	Mismatch		8.5	Match		0.3	Mismatch		0.5	Match
					5.8			9.3			1.7			4.6			0.2			0.7	
1,4-Phenylendiamine	Strong	p	0.9	14.6	18.3		5.6	4.3		16.5	8.4		8.9	3.7		0.0	0.1		0.3	0.3	
					12.1	Match		2.9	Match		14.4	Match		1.6	Mismatch		0.0	Match		0.5	Match
					25.6			9.9			24.1			12.6			0.0			0.4	
Nickel sulfate	Moderate	p	1.1	0.9	1.0		3.6	2.8		2.3	1.5		1.4	0.5		0.6	0.6		0.6	0.6	
					0.7	Match		2.6	Match		2.0	Match		0.3	Mismatch		0.5	Match		0.8	Match
					1.4			4.3			3.5			1.8			0.7			0.8	
2-Mercaptobenzothiazole	Moderate	p	1.1	6.5	7.9		4.5	4.9		12.7	2.2		4.2	0.9		0.1	0.1		0.4	0.6	
					7.2	Match		3.9	Match		27.3	Mismatch		4.6	Mismatch		0.1	Match		0.6	Match
					8.6			6.5			27.7			8.6			0.2			0.6	
R(+)-Limonene	Weak	p	1.1	3.4	3.5		3.9	3.9		8.0	3.6		7.5	2.2		0.1	0.5		0.2	0.5	
					3.5	Match		4.2	Match		44.6	Mismatch		11.6	Mismatch		0.3	Mismatch		0.9	Mismatch
					3.4			8.3			17.9			17.6			0.3			0.7	
Imidazolidinyl urea	Weak	p	1.2	12.0	6.0		19.4	14.5		15.0	12.3		24.3	21.3		0.0	0.0		0.1	0.2	
					5.9	Mismatch		15.8	Match		18.5	Match		11.8	Match		0.0	Match		0.2	Mismatch
					9.3			32.7			13.1			26.7			0.0			0.2	
Isopropanol	non-sensitizer	n	1.0	1.2	1.4		1.2	2.1		1.3	2.1		1.1	1.6		0.8	1.0		1.0	0.9	
					0.7	Match		2.2	Match		3.7	Mismatch		0.8	Match		0.7	Match		1.2	Match
					1.9			2.1			2.8			3.0			1.2			1.1	
Glycerol	non-sensitizer	n	1.0	0.9	1.1		1.1	1.3		1.0	1.0		0.8	0.9		1.2	1.8		0.9	0.7	
					0.7	Match		1.0	Match		1.5	Match		0.4	Match		1.1	Match		1.5	Match
					1.1			2.0			1.9			2.1			1.1			1.1	
4-Aminobenzoic acid	non-sensitizer	n	1.1	1.1	1.0		1.3	0.9		1.1	0.3		1.6	0.4		0.7	0.6		0.9	3.3	
					0.7	Match		0.8	Match		3.8	Match		1.2	Match		0.8	Match		0.9	Match
					0.6			8.2			1.8			2.1			0.5			1.0	

The potency of the sensitizers in the murine LLNA [3,19] is classified as extreme, strong, moderate or weak. In the judgment by h-CLAT [7,26], if either CD86 or CD54 was positive, the test chemical is also judged as positive, “p”. Otherwise, it is indicated by “n”. If the results of new generation sequencing (NGS) and real-time PCR are consistent, it is marked as “Match”; if not, it is marked as “Mismatch”.

**Table 3:** Relative expression levels of candidate marker genes for 28 chemicals.

Chemical	Murine LLNA Category	h-CLAT Judgment	Expression Levels		Judgment	Match or Mismatch with Murine LLNA Category
			*HMOX1*	*JUN*		
Potassium dichromate	Extreme	p	0.9	1.3		
			0.6	1.0	non-sensitizer	Mismatch
			0.4	1.0		
Benzoyl peroxide	Extreme	n	1.9	3.0		
			1.4	2.2	sensitizer	Match
			1.5	2.0		
Cobalt chloride	Strong	p	11.4	1.4		
			18.0	1.4	sensitizer	Match
			18.8	1.2		
4-Nitrobenzyl bromide	Strong	p	92.8	11.8		
			175.7	21.6	sensitizer	Match
			135.9	22.4		
Maleic acid	Strong	p	10.7	1.3		
			11.6	2.0	sensitizer	Match
			13.2	1.3		
2-Aminophenol	Strong	p	2.5	6.7		
			4.2	8.5	sensitizer	Match
			3.2	8.0		
Lauryl gallate	Strong	p	1.7	5.8		
			1.4	7.0	sensitizer	Match
			1.3	6.6		
Methyl methanesulfonate	Moderate	n	3.3	1.6		
			2.3	1.3	sensitizer	Match
			3.6	1.6		
Citral	Moderate	p	220.8	5.9		
			176.9	4.9	sensitizer	Match
			211.8	6.9		
Resorcinol	Moderate	p	1.1	13.6		
			1.2	13.2	sensitizer	Match
			1.7	14.4		
Diethylenetriamine	Moderate	n	19.9	1.8		
			22.3	2.1	sensitizer	Match
			19.9	1.9		
Cinnamaldehyde	Moderate	p	0.7	4.4		
			0.4	3.2	sensitizer	Match
			0.5	3.0		
3-Propylidenephthalide	Moderate	p	135.0	2.4		
			27.0	2.0	sensitizer	Match
			11.0	2.9		
Phenylacetaldehyde	Moderate	p	16.1	3.5		
			18.2	11.7	sensitizer	Match
			29.7	11.0		
3-Dimethylamino propylamine	Moderate	p	79.5	4.9		
			46.6	2.7	sensitizer	Match
			69.7	2.6		
1-Phenyl-1,2-propanedione	Moderate	p	18.2	7.8		
			17.0	7.3	sensitizer	Match
			19.7	6.8		
Isoeugenol	Moderate	n	14.7	3.4		
			13.3	4.3	sensitizer	Match
			19.3	6.3		
Oxalic acid anhydrous	Weak	p	4.6	0.6		
			3.2	0.5	sensitizer	Match
			3.4	0.5		
Geraniol	Weak	p	6.7	13.0		
			8.9	15.2	sensitizer	Match
			7.7	10.3		
1,2-Propanediol	non-sensitizer	n	0.4	0.6		
			0.4	0.6	non-sensitizer	Match
			0.7	0.7		
4-Hydroxybenzoic acid	non-sensitizer	n	0.6	1.0		
			0.7	1.1	non-sensitizer	Match
			0.8	0.8		
Sulfanilamide	non-sensitizer	n	0.8	0.7		
			0.7	0.5	non-sensitizer	Match
			0.9	0.8		
Coumarin	non-sensitizer	n	0.8	8.3		
			0.9	7.8	sensitizer	Mismatch
			1.0	6.3		
4-Methoxyacetophenone	non-sensitizer	n	0.4	1.3		
			0.4	1.9	non-sensitizer	Match
			0.2	1.2		
Ethyl benzoylacetate	non-sensitizer	n	0.9	2.5		
			1.3	3.9	sensitizer	Mismatch
			2.0	11.2		
1-Butanol	non-sensitizer	n	0.7	1.0		
			0.7	0.9	non-sensitizer	Match
			0.9	1.4		
Saccharin	non-sensitizer	n	2.0	0.6		
			1.1	0.4	sensitizer	Mismatch
			2.2	0.8		
Sodium Sulfite	ND	p	0.5	3.5		
			0.4	3.9	sensitizer	-
			0.4	6.5		

The potency of the sensitizers in the murine LLNA [3,27] is classified as extreme, strong, moderate, or weak. In the judgment by h-CLAT [7,26], if either CD86 or CD54 was positive, the test chemical is also judged as positive, “p”. Otherwise, it is indicated by “n”. ND indicates no data.

## Data Availability

Data are available from the corresponding author upon reasonable request.

## Supplementary Materials

The following supporting information can be downloaded at: https://www.mdpi.com/article/10.3390/bios14120632/s1; Table S1: Chemical compounds used in the search for candidate marker genes; Table S2: Chemical compounds used for 5 candidate marker genes; Table S3: Chemical compounds used in the study of the effects on *HMOX1* and *JUN* expression; Table S4: Changes in the gene expression levels in the sensitizing chemical treated group with respect to the non-sensitizing chemical treated group; Table S5: Changes in the gene expression levels in the sensitizing chemical treated group with respect to the control group; Table S6: Transcripts per million (TPM) value for 13 genes; Table S7: Relative expression levels of candidate marker genes for 9 chemicals; Table S8: Sixty-two genes whose expression levels were altered in the sensitizing chemical treatment compared to the non-sensitizing treatment; Table S9: Sixty-two genes whose expression levels were altered in the sensitizing chemical treatment compared to the medium treatment. Reference [42] is cited in the supplementary materials.
